# Retraction: Methylation-mediated silencing of microRNA-211 promotes cell growth and epithelial to mesenchymal transition through activation of the AKT/β-catenin pathway in GBM

**DOI:** 10.18632/oncotarget.28840

**Published:** 2026-04-24

**Authors:** Weidong Li, Xiaobo Miao, Lingling Liu, Yue Zhang, Xuejun Jin, Xiaojun Luo, Hai Gao, Xubin Deng

**Affiliations:** ^1^Affiliated Cancer Hospital of Guangzhou Medical University, Guangzhou, China; ^2^Department of Radiation and Chemotherapy Oncology, Ningbo No.2 Hospital, Ningbo, China; ^3^Department of Hematology, The Third Affiliated Hospital of Sun Yat-Sen University, Guangzhou, China; ^4^Department of Radiation Oncology, The First Affiliated Hospital, Zhengzhou University, Zhengzhou, China; ^5^Traditional Chinese Medicine-Integrated Hospital of Southern Medical University, Guangzhou, China; ^6^Xiamen Hospital of Traditional Chinese Medicine, Xiamen, China; ^7^Xiamen Hospital Affiliated to Fujian University of Traditional Chinese Medicine, Xiamen, China; ^*^These authors have contributed equally to this work

**This article has been retracted:** Oncotarget has completed its investigation of this paper. It was determined that the article contains instances of image duplication and overlap with unrelated papers:

- Figure 1A: The SNAP25 blot is a duplicate of the GAPDH blot in Figure 5B of earlier published paper [1].
- Figure 3A: Five tumor images are duplicates of tumor images in Figure 6B from earlier submitted unrelated paper [2].
- Figure 5A: The p-Akt blot was found to be identical to the p-Akt blot in Figure 5B of [2].
- Figure 4D: The GAPDH image was found in Figure 4F as b-actin in later publication [3].

The authors were notified of these findings. While the corresponding author, Dr. Deng, promised to clarify the issue, no response was provided. Given the nature of these duplications, the Editorial decision has been made to retract the article. Oncotarget has reached out to all authors, and they have expressed their agreement with this retraction. Additionally, the authors identified that the U87 glioma cell line used in this study may have been cross-contaminated with other cell lines. While this is separate from the duplication allegations, it further affects the confidence in the reproducibility and completeness of the experimental data.

Original article: Oncotarget. 2017; 8:25167–25176. 25167-25176. https://doi.org/10.18632/oncotarget.15531
